# Some Remarks upon the Efficacy of General Remedies, and Especially Purgatives, in Insuring a Healthy State of the Urinary Secretion, and Thus in Preventing Calculous Affections

**Published:** 1818-06

**Authors:** 


### *Some Remarks upon the Efficacy of general Remedies, and especially Purgatives, in insuring a healthy State of the Urinary Secretion, and thus in preventing Calculous Affections.* By WILLIAM PROUT, M.D.

[ Medico-Chirurg. Transactions, vol. viii. ]

DR. Prout's attention to the subject under consideration was excited, several years ago, from noticing the effect of purgatives on the urinary secretion in his own person; namely, that of restoring it from an unnatural and turbid state to its proper colour and transparency. The natural conclusion which he drew was, that " the cause, whatever it might be, which rendered laxatives necessary, contributed chiefly to produce this unhealthy state of the urine." Aware of the affinity between urinary deposits and urinary concretions, our author naturally queries whether, if purgatives have the power of removing urinary sediments in common cases, they may not also have the power of removing them in extreme cases, or in gravelly or calculous affections?

" Vitiated secretions (says Dr. Prout), of every description, must be the result of general or of local causes, or of both united. But when we reflect how little liable the secreting organs are to be affected, and how seldom, in point of fact, they are affected, except through the medium of the general health, we are naturally led to look here for the primary cause of their derangement. The inference is obvious. Remedies, no matter of what description, that have a tendency to restore the general health, must have a tendency to insure the due performance of all the bodily functions, and secretion among the rest. I need not enlarge here upon principles which are well understood, and the elucidation and application of which are justly ranked among the greatest discoveries of modern medicine; but shall merely observe, that by paying proper attention to the general health, and especially to the functions of the stomach and bowels, I have, in numerous instances, witnessed the speedy removal of urinary deposits, and the complete restoration of this secretion to its natural appearance and properties. This has been remarkably the case in children, in whom the phosphoric diathesis most generally prevails. The remedy employed has been for the most part a combination of rhubarb and calomel, in connection with which others were occasionally exhibited as circumstances rendered it necessary. In adults, as is well known, both the phosphoric and lithic diatheses prevail, and often alternate in the same person. I have, however, generally seen both equally yield to the same principles of treatment, and sometimes even to the same remedy; and am disposed to think, that they are more intimately connected than commonly imagined. Some differences, however, must be admitted to exist between causes which can produce such different effects, though I must confess myself unable, after a good deal of attention to the subject, to point out, or even to offer an opinion of their nature. As to particular remedies, they will readily occur to the practitioner who keeps the above-mentioned principles in view. I may, however, observe, that when laxatives have been particularly indicated, I have been accustomed to exhibit a combination of pil. hyd. with aloes, or the ex. col. com. with the best effect. Remedies determining to the skin and kidnies are often useful adjuncts, and a regimen in strict unison with the same general principle should be adopteds." P. 546.

The above observations are confined, of course, to diseases of the urine, while as yet they are merely constitutional, and have not produced local disease or actual calculus. To the treatment of this last class of human ailments Dr. Prout has nothing to add.

Dr. P. judiciously observes, that the good effects of acid and alkaline remedies cannot be satisfactorily explained on *chemical* principles. The propensity to generate lithic or phosphoric acid is so capricious, and so frequently alternates in the same person, and under *apparently* similar circumstances, that, reasoning *chemically* upon the subject, the exhibition of acid or alkaline remedies is as likely to do harm as good. And, after all, the *object* of the chemical practitioner is, at best, but of a secondary importance. It is to prevent the effects of disease, rather than to remove it. Chemical remedies are therefore only palliatives; and it is highly probable, as Dr. Prout observes, that their good effects depend more on their general than their chemical operation.

Dr. Prout is evidently a man of comprehensive ideas and clear judgment. The paper is equally creditable to his professional skill and his personal modesty.

